# Dinaciclib Interrupts Cell Cycle and Induces Apoptosis in Oral Squamous Cell Carcinoma: Mechanistic Insights and Therapeutic Potential

**DOI:** 10.3390/ijms26052197

**Published:** 2025-02-28

**Authors:** Muhammet Oner, Yu-Chiao Cheng, Shiuan-Woei Soong, Pang-Ting Cheng, Yan-Hsiung Wang, Shun-Fa Yang, Stella Chin-Shaw Tsai, Ho Lin

**Affiliations:** 1 Department of Life Sciences, National Chung Hsing University, Taichung 40227, Taiwan; muhammet.oner053@gmail.com (M.O.); sophia2919@gmail.com (Y.-C.C.); tommy86921@gmail.com (S.-W.S.); iq4153333@gmail.com (P.-T.C.); 2 Translational Cell Therapy Center, Department of Medical Research, China Medical University Hospital, Taichung 40447, Taiwan; 3 School of Dentistry, Kaohsiung Medical University, Kaohsiung 807378, Taiwan; yhwang@kmu.edu.tw; 4 Department of Medical Research, Chung Shan Medical University Hospital, Taichung 40201, Taiwan; ysf@csmu.edu.tw; 5 Institute of Medicine, Chung Shan Medical University, Taichung 40201, Taiwan; 6 Superintendent Office, Tungs’ Taichung MetroHarbor Hospital, Taichung 43503, Taiwan; 7 College of Life Sciences, National Chung Hsing University, Taichung 40227, Taiwan; 8 Department of Post-Baccalaureate Medicine, National Chung Hsing University, Taichung 40227, Taiwan

**Keywords:** Dinaciclib, cell cycle arrest, cyclin-dependent kinases (CDKs), oral squamous cell carcinoma (OSCC)

## Abstract

Dinaciclib, a potent cyclin-dependent kinase (CDK) inhibitor, has demonstrated considerable antitumor effects in various malignancies. However, its impact on oral squamous cell carcinoma (OSCC), a predominant and highly aggressive form of head and neck squamous cell carcinoma (HNSC) with limited treatment options, remains underexplored. We conducted gene set enrichment analyses in HNSC patients that reinforced the relevance of these cell cycle-related genes to OSCC pathogenesis. Given the known dysregulation of cell cycle-related genes in HNSC patients, we hypothesized that Dinaciclib may inhibit OSCC growth by targeting overexpressed cyclins and CDKs, thereby disrupting cell cycle progression and inducing apoptosis. This study investigated Dinaciclib’s effects on cell proliferation, cell cycle progression, and apoptosis in the OSCC cell lines Ca9-22, OECM-1, and HSC-3. Our results demonstrated that Dinaciclib significantly reduces OSCC cell proliferation in a dose-dependent manner. Flow cytometry and Western blot analyses showed that Dinaciclib induces cell cycle arrest at the G1/S and G2/M transitions by downregulating Cyclins A, B, D, and E, along with CDKs 1 and 2—key regulators of these checkpoints. Furthermore, Dinaciclib treatment upregulated apoptotic markers, such as cleaved-caspase-3 and cleaved-PARP, confirming its pro-apoptotic effects. In conclusion, these findings highlight Dinaciclib’s therapeutic promise in OSCC by simultaneously disrupting cell cycle progression and inducing apoptosis. These results support further exploration of Dinaciclib as a viable monotherapy or combination treatment in OSCC and other HNSC subtypes to improve patient outcomes.

## 1. Introduction

Oral squamous cell carcinoma (OSCC) is a major subtype of head and neck squamous cell carcinoma (HNSC), which includes cancers arising in the oral cavity, pharynx, and larynx. HNSC is known for its aggressive nature, high recurrence rates, and overall poor prognosis, especially in advanced stages [1,2]. Like other forms of HNSC, OSCC is characterized by its rapid proliferation and metastatic potential, with common risk factors including tobacco use, alcohol consumption, and human papillomavirus (HPV) infection [3]. The relationship between OSCC and HNSC is particularly important for understanding both disease mechanisms and treatment options. As a subtype within the broader HNSC category, OSCC shares many therapeutic targets, and findings in HNSC research often apply to OSCC as well, underscoring the relevance of targeted therapies, such as CDK inhibitors, that aim to disrupt the cell cycle in these malignancies. Despite advances in surgical, chemotherapeutic, and radiotherapeutic interventions, the prognosis for OSCC remains poor, with 5-year survival rates barely exceeding 50% in advanced cases [4]. High rates of recurrence, metastasis, and therapeutic resistance in OSCC underscore an urgent need for novel and effective treatment strategies [2,5,6]. Central to the pathology of OSCC is dysregulated cell cycle control, which promotes unchecked cellular proliferation and tumor progression [7,8]. Cyclin-dependent kinases (CDKs), as primary regulators of cell cycle transitions, represent a promising therapeutic target for controlling cancer cell proliferation in OSCC [9,10,11,12,13].

CDKs regulate cell cycle progression through tightly controlled interactions with their partner proteins, cyclins [14,15]. The cell cycle is divided into four distinct phases: G1, S, G2, and M, each regulated by specific cyclin–CDK complexes. For instance, the G1/S transition is controlled by the Cyclin D–CDK4/6 and Cyclin E–CDK2 complexes, while the S and G2/M phases are driven by Cyclin A–CDK2 and Cyclin B–CDK1 complexes, respectively [16,17]. Dysregulation of these complexes, which can be due to overexpression or mutations in cyclins and CDKs, has been observed in OSCC, where it contributes to continuous cell cycle progression and tumor growth [10].

Dinaciclib, a potent inhibitor of multiple CDKs, has garnered considerable attention in recent years for its antiproliferative and pro-apoptotic effects across various malignancies, including breast cancer [18], leukemia [19], and lung cancer [20]. By selectively targeting CDKs involved in both cell cycle regulation and transcriptional control, Dinaciclib disrupts critical checkpoints in the cell cycle, thereby halting tumor cell proliferation [21]. Inhibiting CDKs 1 and 2, which are essential for the G1/S and G2/M transitions, effectively impedes cellular entry into the S phase and mitosis, leading to cell cycle arrest [22,23]. Additionally, CDK5 and CDK9 play roles in transcriptional regulation and apoptosis [22,23,24]; thus, their inhibition by Dinaciclib can enhance cancer cell susceptibility to programmed cell death. Moreover, recent findings indicate that Dinaciclib can induce apoptosis through the activation of caspase pathways and cleavage of PARP, both markers of programmed cell death [25,26]. This is particularly relevant to OSCC, which is often resistant to apoptosis due to dysregulated apoptotic pathways. In the context of OSCC, the ability of Dinaciclib to overcome these apoptotic barriers by directly targeting CDKs could make it an effective treatment option, especially for patients with tumors resistant to conventional therapies [27].

While promising, the effects of Dinaciclib on OSCC have not been well documented, and the potential of CDK inhibition as a treatment strategy for OSCC remains largely unexplored. This study aims to investigate Dinaciclib’s impact on cell cycle regulation and apoptosis induction in OSCC, with the hypothesis that its multi-CDK inhibitory action would result in robust cell cycle arrest and apoptosis in OSCC cells. Understanding Dinaciclib’s effect on cell cycle-related proteins, including cyclins and CDK inhibitors, is crucial in treating OSCC and could contribute to halting disease progression. We therefore hypothesize that Dinaciclib will induce significant changes in the expression of these cyclins, resulting in cell cycle arrest at critical transition points in the OSCC cell cycle. Thus, the objective of this study is two-fold: it aims to assess Dinaciclib’s capacity to induce cell cycle arrest and apoptosis in OSCC cell lines and to explore its effects on the regulation of key cell cycle proteins, including Cyclins A, B, D, and E; CDKs 1 and 2; and the CDK inhibitors p21 and p27. By elucidating these mechanisms, we aim to provide a deeper understanding of Dinaciclib’s therapeutic potential in OSCC and contribute to the development of more effective treatment strategies for this challenging malignancy.

## 2. Results

### 2.1. Upregulation of Cell Cycle-Related Genes in HNSC Tumors Compared to Normal Tissues

**Figure 1:**
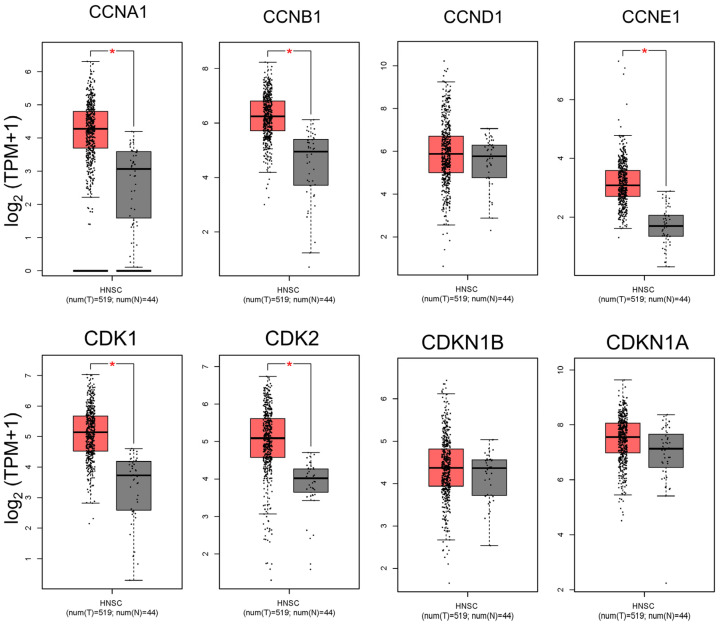
Comparison of cell cycle-related gene expression between HNSC tumors and normal tissues. This figure presents a differential expression analysis of key cell cycle-related genes between head and neck squamous cell carcinoma (HNSC) tumor samples and adjacent normal tissues. The genes analyzed include Cyclins A, B, D, and E and CDKs 1 and 2, which are critical for cell cycle regulation and progression through the G1/S and G2/M transitions. The data reveal significant upregulation of these genes in HNSC tumors relative to normal tissues, indicating enhanced cell cycle activity and potential dysregulation in tumor cells. Elevated expression of Cyclin D1, Cyclin E1, and CDK2 supports increased G1/S transition activity, which may facilitate the high proliferation rate characteristic of aggressive cancers like HNSC. Similarly, the upregulation of Cyclin B1 and CDK1 suggests increased G2/M transition activity, emphasizing the role of these genes in the unchecked growth and survival of HNSC cells. (* *p* < 0.05 compared to the control).

To investigate cell cycle dysregulation in head and neck squamous cell carcinoma (HNSC), we analyzed the expression profiles of several key cell cycle-related genes in HNSC tumors compared to adjacent normal tissues (Figure 1). Our analysis focused on cyclins (CCNA1, CCNB1, CCND1, and CCNE1) and cyclin-dependent kinases (CDKs, including CDK1 and CDK2), which are critical for the regulation of cell cycle progression. Cyclin A1 (CCNA1) and Cyclin B1 (CCNB1) are particularly essential for controlling the S phase and G2/M transition, respectively, while Cyclin D1 (CCND1) and Cyclin E1 (CCNE1) drive progression through the G1/S transition. CDKs, specifically CDK1 and CDK2, partner with these cyclins to facilitate phase-specific checkpoints in the cell cycle. Our findings reveal that these genes are significantly upregulated in HNSC tumor tissues compared to normal tissues, suggesting enhanced cell cycle activity within the tumor environment (Figure 1). Elevated levels of Cyclins D1 and E1, along with CDK2, align with increased G1/S transition activity, which may contribute to the uncontrolled proliferation characteristic of HNSC. Similarly, the upregulation of Cyclins A1 and B1, along with CDK1, points to heightened activity at the S phase and G2/M transition, stages that are critical for DNA replication and mitosis (Figure 1). This pervasive upregulation of cell cycle-related genes in HNSC tumors highlights the dysregulated cell cycle control that underpins tumor growth and supports the rationale for targeting these proteins therapeutically in OSCC and HNSC.

### 2.2. Enrichment of Cell Cycle Pathways and Correlation with Tumor Cell Cycle Regulation in HNSC Patients

**Figure 2:**
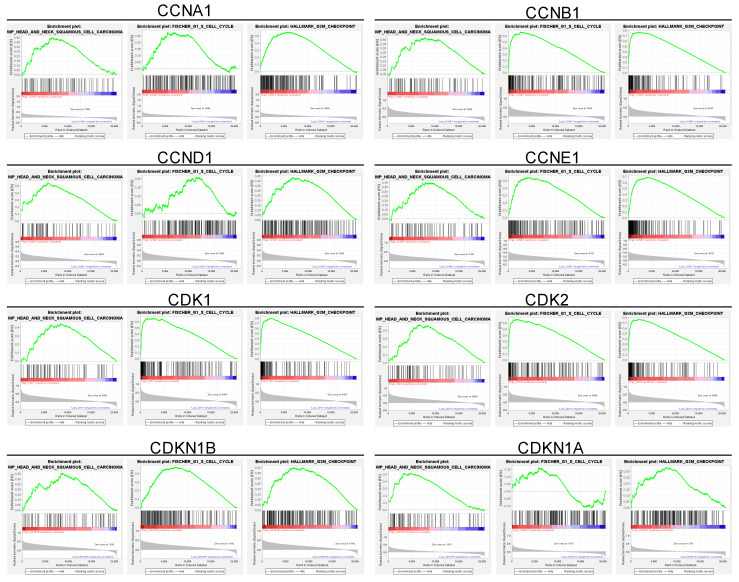
Gene set enrichment analysis of cell cycle-related genes and their correlation with cell cycle regulation in HNSC patients. This figure highlights enriched pathways associated with cell cycle regulation. The analysis identifies a significant association between upregulated cell cycle genes and critical processes, including the G1/S and G2/M transitions, as well as DNA replication. These enriched pathways underscore the role of cell cycle dysregulation in HNSC tumorigenesis, with implications for accelerated cell division and genomic instability. Specifically, enrichment of the G1/S transition pathway aligns with elevated Cyclin D1, Cyclin E1, and CDK2 levels, which drive cells into the S phase, enhancing DNA synthesis and proliferation. Similarly, enrichment of the G2/M transition pathway, associated with Cyclin B1 and CDK1, facilitates rapid entry into mitosis, supporting high tumor cell turnover. This pathway enrichment provides further rationale for targeting cell cycle dysregulation in HNSC, supporting the therapeutic potential of CDK inhibitors such as Dinaciclib. Red indicates high gene set enrichment, while blue represents low enrichment.

To further elucidate the relationship between these upregulated genes and cell cycle dysregulation in HNSC, we conducted a gene set enrichment analysis (GSEA) of the cell cycle-related gene set in HNSC patients (Figure 2). The GSEA revealed that the upregulated cell cycle-related genes were strongly correlated with pathways governing key cell cycle processes, including DNA replication, G1/S transition, and G2/M transition checkpoints (Figure 2). These enriched pathways underscore the critical role of cell cycle dysregulation in HNSC, implicating the upregulated genes in the enhanced cell division and genomic instability observed in tumor cells. Specifically, the enrichment of the G1/S transition pathway is associated with elevated levels of Cyclin D1, Cyclin E1, and CDK2, which together regulate the cell’s entry into the S phase, initiating DNA synthesis (Figure 2). This process is crucial for maintaining a high proliferation rate, which is characteristic of aggressive tumors like HNSC. Additionally, enrichment of G2/M transition pathways suggests that increased expression of Cyclin B1 and CDK1 accelerates the cell’s entry into mitosis, further supporting the rapid cellular turnover observed in HNSC tumors (Figure 2). In summary, these findings indicate that the cell cycle-related genes upregulated in HNSC tumors are closely associated with enriched pathways controlling DNA replication, G1/S transition, and G2/M transition checkpoints, suggesting a crucial role for these genes in driving tumor cell proliferation and genomic instability in HNSC.

### 2.3. Dinaciclib Inhibits Oral Squamous Cell Carcinoma (OSCC) Cell Proliferation in a Dose-Dependent Manner

**Figure 3:**
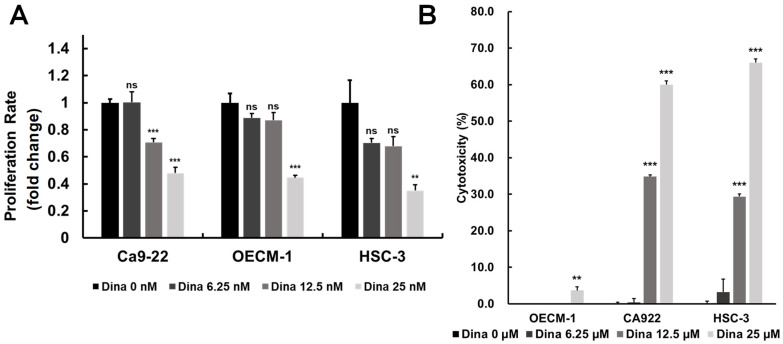
Dinaciclib inhibits cell proliferation and induces cytotoxicity in OSCC cell lines. (**A**) OSCC cell lines (Ca9-22, OECM-1, and HSC-3) were treated with varying concentrations of Dinaciclib (0, 6.25, 12.5, and 25 nM) for 24 h. Cell viability was assessed via the MTT assay, which showed a dose-dependent decrease in cell proliferation across all cell lines, with significant reductions at 12.5 nM and 25 nM. Ca9-22 cells showed a notable reduction in viability at 12.5 nM, while OECM-1 and HSC-3 cells required the highest concentration (25 nM) for significant inhibition. Data are presented as mean ± SD values; statistical significance was determined using an unpaired Student’s *t*-test (** *p* < 0.01, and *** *p* < 0.001 compared to the control). (**B**) The cytotoxic effects of Dinaciclib were evaluated using an LDH assay, which quantifies LDH release as an indicator of cell membrane integrity loss. Dinaciclib treatment led to increased cytotoxicity at higher concentrations across all OSCC cell lines, supporting its dose-dependent impact on reducing cell viability. Statistical analysis was conducted using an unpaired Student’s *t*-test (** *p* < 0.01, and *** *p* < 0.001 compared to the control).

Oral squamous cell carcinoma (OSCC) is a common and aggressive malignancy characterized by rapid growth, resistance to conventional therapies, and high recurrence rates. A key driver of OSCC progression is the dysregulation of cell cycle control, primarily through the overactivation of cyclin-dependent kinases (CDKs) (Figure 1 and Figure 2), which promote uncontrolled cell proliferation [28]. By inhibiting upregulated cyclins and CDKs, it may be possible to disrupt key checkpoints in the cell cycle, slowing tumor growth and reducing proliferation rates. The above findings are particularly relevant to the exploration of CDK inhibitors, such as Dinaciclib, which targets multiple CDKs (including CDK1 and CDK2) and has shown promising effects in inducing cell cycle arrest and apoptosis in OSCC. Therefore, we investigated the effects of Dinaciclib on OSCC proliferation and cytotoxicity. Treatment with Dinaciclib demonstrated a dose-dependent effect on cell viability across the OSCC cell lines Ca9-22, OECM-1, and HSC-3 (Figure 3A). Treatment with 6.25 nM Dinaciclib did not significantly affect cell proliferation in any of the OSCC cell lines. At 12.5 nM, cell viability decreased significantly only in Ca9-22 cells, with no notable changes in the OECM-1 or HSC-3 cells. At the highest concentration (25 nM), Dinaciclib significantly reduced cell viability across all cell lines, reaching up to 80% inhibition in the Ca9-22 and HSC-3 cells and nearly 70% in the OECM-1 cells (Figure 3A). In addition, Dinaciclib significantly induced cellular cytotoxicity at concentrations above 12.5 nM across all OSCC cell lines (Figure 3B). These findings indicate that while lower concentrations of Dinaciclib have a limited impact on OSCC proliferation, higher concentrations produce a pronounced antiproliferative effect by enhancing cellular cytotoxicity. This dose-dependent inhibition highlights Dinaciclib’s potential as a therapeutic agent in OSCC as it effectively reduces cell viability at clinically relevant concentrations.

### 2.4. Dinaciclib Induces Cell Cycle Arrest in OSCC Cells

**Figure 4:**
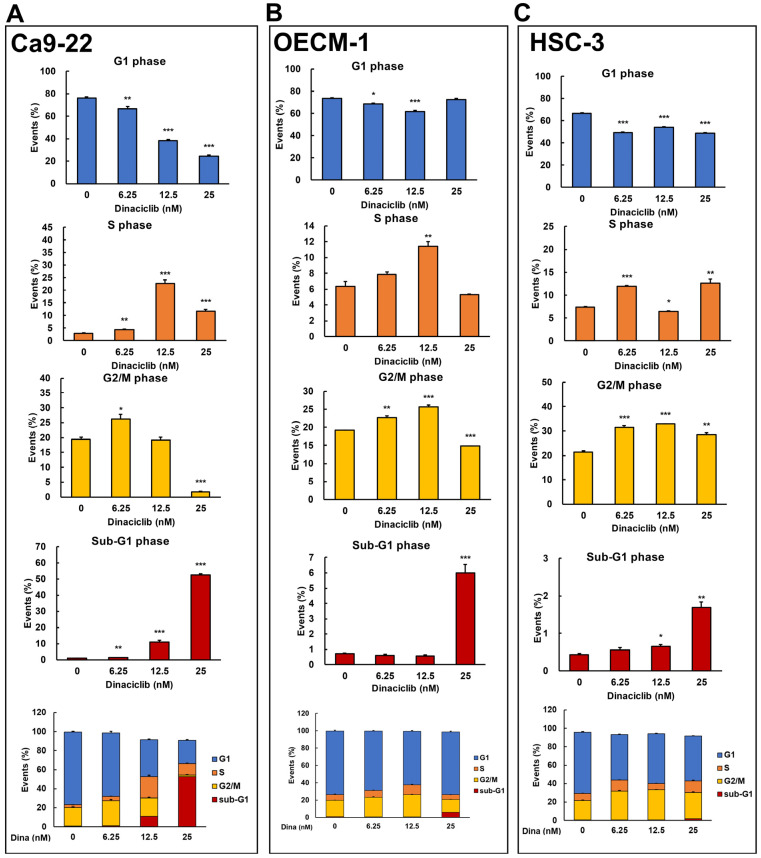
Dinaciclib induces cell cycle arrest at specific phases in OSCC cell lines. OSCC cell lines were treated with Dinaciclib at concentrations of 0, 6.25, 12.5, and 25 nM for 24 h, followed by cell cycle analysis via flow cytometry to determine phase-specific arrest. (**A**) Ca9-22 cells exhibited a dose-dependent S phase arrest, with an increased S phase population at 12.5 nM and further accumulation at 25 nM. A marked increase in sub-G1 phase cells was observed at 25 nM, indicative of apoptosis. (**B**) In OECM-1 cells, Dinaciclib induced dual-phase arrest, significantly increasing both S and G2/M phase populations at higher concentrations (12.5 and 25 nM). Sub-G1 accumulation at 25 nM confirmed apoptosis induction. (**C**) HSC-3 cells showed strong G2/M phase arrest at 25 nM, along with an increase in sub-G1 cells, which is consistent with apoptotic cell death. Statistical significance was determined using an unpaired Student’s *t*-test (* *p* < 0.05, ** *p* < 0.01, *** *p* < 0.001 compared to the control).

Dinaciclib is a potent and selective inhibitor of multiple cyclin-dependent kinases (CDKs), which play critical roles in cell cycle progression and transcriptional regulation [21]. Previous studies have demonstrated that Dinaciclib can induce cell cycle arrest and apoptosis in various cancers, including breast and lung cancers, by disrupting CDK-driven pathways [18,20]. However, its effects on cell cycle arrest in OSCC cells remain unclear. To address this, we analyzed Dinaciclib-mediated cell cycle events in OSCC cell lines in a dose-dependent manner. Flow cytometry revealed that Dinaciclib treatment significantly arrested the cell cycle in OSCC cells, with variations in the degree and phase of arrest depending on the cell line and concentration used. In Ca9-22 cells, Dinaciclib induced a dose-dependent S phase arrest, with a clear increase in the S phase population as concentrations increased (Figure 4A and Table 1). Additionally, a marked increase in the sub-G1 population was observed at higher concentrations, indicating that apoptosis was induced in the Ca9-22 cells (Figure 4A and Table 1). In the OECM-1 cells, Dinaciclib treatment led to a notable accumulation in both the S and G2/M phase populations at increasing concentrations, suggesting dual-phase arrest in this cell line (Figure 4B and Table 2). At the highest concentration, there was also an increase in the sub-G1 population, which is consistent with apoptotic cell death (Figure 4B and Table 2). Similarly, in HSC-3 cells, Dinaciclib induced a prominent accumulation in the G2/M phase, with additional S phase arrest at higher concentrations, indicating that Dinaciclib effectively halts cell cycle progression in this cell line as well (Figure 4C and Table 3). An increase in the sub-G1 population was also observed, further supporting apoptosis induction in HSC-3 cells (Figure 4C and Table 3). These results suggest that Dinaciclib’s cell cycle inhibitory effects vary slightly by cell line yet consistently result in S and/or G2/M phase arrest and apoptosis, underscoring its potential as a therapeutic agent for targeting dysregulated cell cycle pathways in OSCC.

### 2.5. Dinaciclib Modulates Key Cell Cycle- and Apoptosis-Related Proteins in OSCC

**Figure 5:**
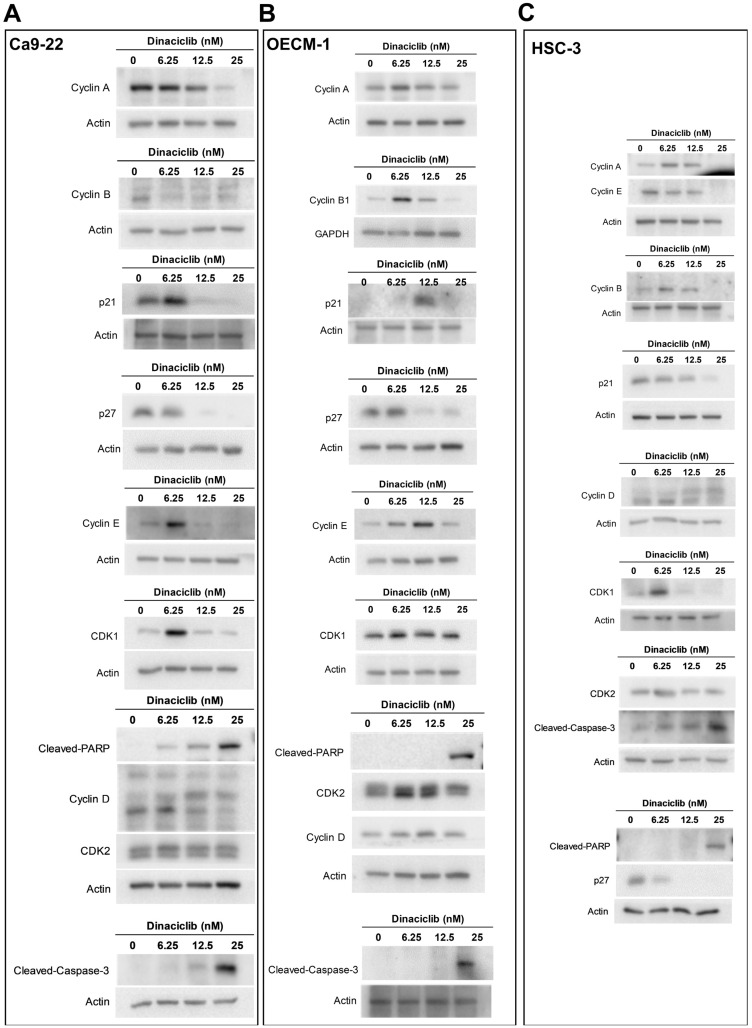

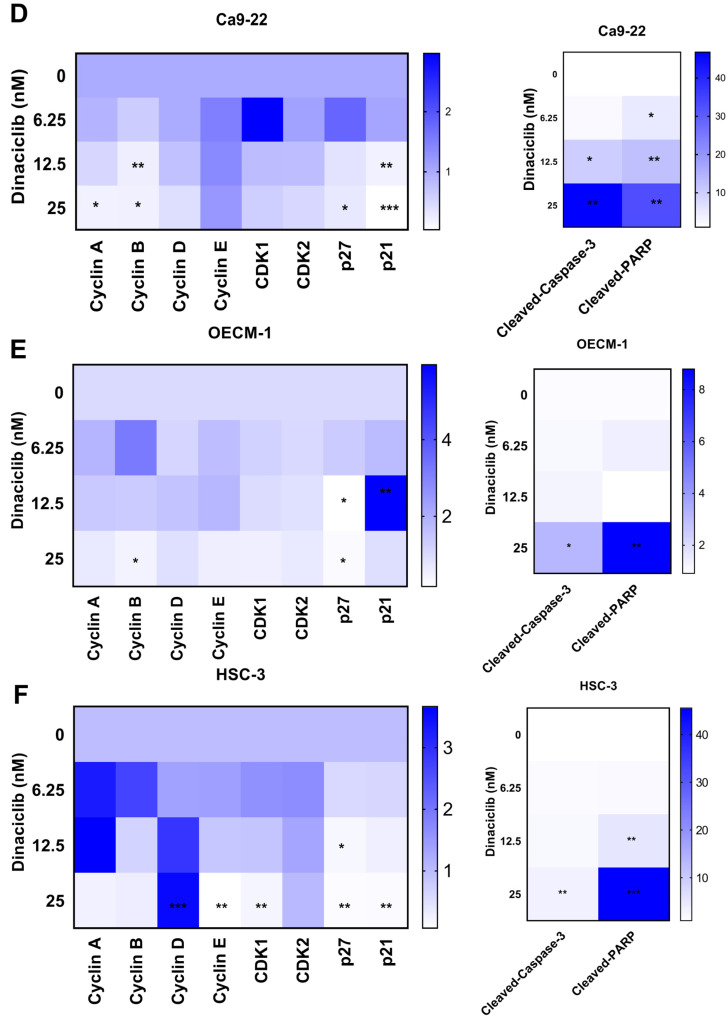
Dinaciclib downregulates key cell cycle regulators and induces apoptotic markers in OSCC cells. OSCC cell lines (Ca9-22, OECM-1, and HSC-3) were treated with Dinaciclib at 0, 6.25, 12.5, and 25 nM for 24 h, and protein expression was analyzed via Western blotting and heatmap visualization. (**A**–**C**) Western blot analysis of cell cycle-related proteins and apoptotic markers in Ca9-22 (**A**), OECM-1 (**B**), and HSC-3 (**C**) cells. Dinaciclib treatment led to a dose-dependent decrease in the expression of Cyclins A, B, D, and E, as well as CDKs 1 and 2, across all cell lines. Additionally, cleaved-caspase-3 and cleaved-PARP were significantly upregulated, confirming apoptosis induction. (**D**–**F**) Heatmaps representing the relative expression levels of the proteins quantified in panels A, B, and C for the Ca9-22 (**D**), OECM-1 (**E**), and HSC-3 (**F**) cells, respectively. The heatmaps show a graded reduction in Cyclins A, B, D, and E, CDKs 1 and 2, p21, and p27, along with a dose-dependent increase in cleaved-caspase-3 and cleaved-PARP, supporting the dual mechanism of cell cycle arrest and apoptosis induced by Dinaciclib in OSCC cells. Statistical significance was determined using an unpaired Student’s *t*-test (* *p* < 0.05, ** *p* < 0.01, *** *p* < 0.001 compared to the control).

After establishing that Dinaciclib induces cell cycle arrest and a significant increase in the sub-G1 phase, which is indicative of apoptosis in OSCC, we further investigated its specific effects on cell cycle-related proteins and apoptotic markers through Western blot analysis using three OSCC cell lines (Ca9-22, OECM-1, and HSC-3). Western blot analysis revealed that Dinaciclib downregulated several key cell cycle regulators in a dose-dependent manner, including Cyclins A, B, D, and E, as well as CDKs 1 and 2, across all three OSCC cell lines (Figure 5A–C).

In more detail, the expression levels of Cyclins A and B, which are primarily involved in regulating the S and G2/M phases of the cell cycle, respectively [29], were significantly reduced following Dinaciclib treatment (Figure 5D–F). In Ca9-22 cells, Cyclin A levels dropped markedly, corresponding with the S phase arrest observed in this cell line (Figure 5A,D). Similarly, Cyclin B, a crucial regulator of the G2/M transition, was downregulated in the OECM-1 and HSC-3 cells at higher Dinaciclib concentrations, aligning with the G2/M arrest observed in these cell lines (Figure 5B,E). The decrease in Cyclin B suggests that Dinaciclib disrupts progression through the G2/M checkpoint, further contributing to cell cycle arrest. Cyclins E, which regulate the G1/S transition, were also notably decreased in the OECM-1 and HSC-3 cell lines with increasing Dinaciclib concentrations (Figure 5E,F). Reduced Cyclin E levels in cells additionally correlate with S phase arrest in these cell lines, reinforcing Dinaciclib’s impact on halting the initiation of DNA replication.

Consistent with the increase in the sub-G1 population observed in all OSCC cell lines, Dinaciclib induced apoptosis, as evidenced by the upregulation of cleaved-caspase-3 and cleaved-PARP, markers of apoptotic cell death (Figure 5A–F). The activation of caspase-3, an essential effector in the apoptosis cascade, was most pronounced at the highest concentration of Dinaciclib across all three cell lines. The increase in cleaved-PARP, a downstream target of caspase-3, further supports the conclusion that Dinaciclib induces apoptosis following cell cycle arrest. In summary, Dinaciclib disrupts cell cycle progression in OSCC cells by downregulating cyclins and CDKs associated with both the G1/S and G2/M transitions. The concurrent induction of apoptosis through caspase-3 and PARP cleavage underscores Dinaciclib’s dual mechanism of action in OSCC cells, supporting its potential as a therapeutic agent capable of targeting dysregulated cell cycle pathways and promoting cell death in OSCC.

**Figure 6:**
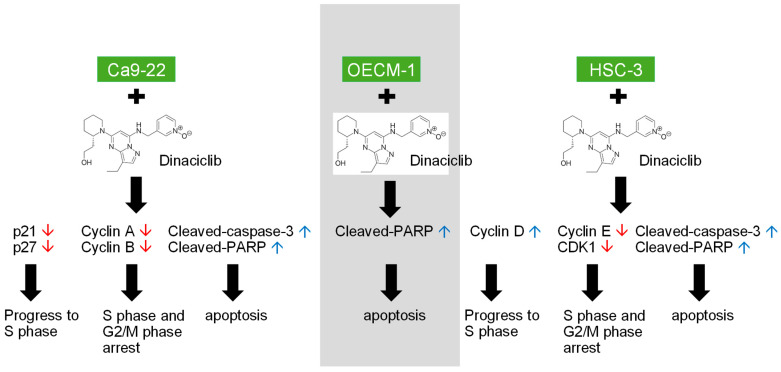
Dinaciclib, a potent CDK inhibitor, demonstrates substantial antitumor effects in OSCC cell lines (Ca9-22, OECM-1, and HSC-3) by targeting multiple cell cycle and apoptotic pathways. This graphical abstract illustrates the two main effects of Dinaciclib treatment on OSCC cells. (1) Cell Cycle Arrest: Dinaciclib downregulates the expression of Cyclins A, B, D, and E, along with CDKs 1 and 2, effectively halting cell cycle progression at the G1/S and G2/M phases. (2) Apoptosis Induction: Dinaciclib promotes apoptosis, evidenced by the upregulation of cleaved-caspase-3 and cleaved-PARP. This apoptotic effect correlates with increased sub-G1 populations, indicating cell death and cytotoxicity. This study underscores Dinaciclib’s potential as a therapeutic agent for OSCC by disrupting CDK activity, enforcing cell cycle arrest, and promoting apoptosis. Red arrows indicate downregulation, while blue arrows indicate upregulation.

## 3. Discussion

Our study investigates the potential of Dinaciclib, a CDK inhibitor, as a therapeutic agent for oral squamous cell carcinoma (OSCC), a major subtype of head and neck squamous cell carcinoma (HNSC) known for its aggressive behavior and limited treatment options. We selected the Ca9-22, OECM-1, and HSC-3 cell lines to represent the genetic, phenotypic, and cell cycle diversity of OSCC, providing a comprehensive model for our study. Ca9-22 is derived from gingival carcinoma, OECM-1 from oral epithelial carcinoma, and HSC-3 from a metastatic OSCC site. These cell lines differ in p53 status, proliferation rate, metastatic potential, and cell cycle regulation. Specifically, Ca9-22 exhibits a relatively stable cell cycle with moderate proliferation, OECM-1 shows altered cell cycle checkpoints with increased S-phase progression, and HSC-3 displays aggressive growth with disrupted G1/S regulation, which is characteristic of metastatic OSCC. This diversity allows us to investigate various aspects of OSCC pathogenesis, increasing the relevance of our findings to the broader OSCC context [30,31].

Dinaciclib is a broad-spectrum CDK inhibitor targeting CDK1, CDK2, CDK5, and CDK9, which enables it to induce cell cycle arrest at multiple checkpoints (G1/S and G2/M) and trigger apoptosis through the transcriptional suppression of anti-apoptotic proteins [25]. This multi-target approach contributes to its potent anti-cancer activity but also accounts for its higher toxicity profile compared to more selective inhibitors. In contrast, CDK4/6 inhibitors such as Palbociclib, Ribociclib, and Abemaciclib selectively target CDK4 and CDK6, primarily inducing G1 phase arrest by inhibiting the phosphorylation of the retinoblastoma (Rb) protein [32,33,34]. While these inhibitors have shown significant clinical efficacy in hormone receptor-positive breast cancers, their therapeutic effects in OSCC appear limited, likely due to the complex molecular landscape and compensatory pathways active in head and neck cancers. Moreover, CDK4/6 inhibitors are generally better tolerated, with fewer hematological toxicities compared to Dinaciclib, although they can still cause neutropenia and gastrointestinal side effects [35].

Other pan-CDK inhibitors, such as Flavopiridol, also target multiple CDKs but differ in their potency and toxicity profiles. Dinaciclib has demonstrated superior efficacy and a more favorable pharmacokinetic profile than Flavopiridol in preclinical studies and early-phase clinical trials [36]. Additionally, Dinaciclib’s inhibition of CDK9 disrupts transcriptional elongation, leading to the rapid depletion of short-lived anti-apoptotic proteins, a mechanism not prominently targeted by CDK4/6 inhibitors [24]. Overall, while CDK4/6 inhibitors offer selective targeting with manageable toxicity, Dinaciclib’s broader inhibition spectrum provides more robust anti-cancer effects, particularly in OSCC, in which multiple CDK-related pathways contribute to tumor progression. However, this advantage comes at the cost of increased toxicity, underscoring the need for careful dose optimization and potential combination therapies to maximize efficacy while minimizing adverse effects. This comparative analysis highlights Dinaciclib’s unique advantages and challenges, emphasizing its potential role in OSCC treatment strategies.

The findings of our research highlight the dysregulation of cell cycle-related genes in HNSC tumors, particularly the upregulation of Cyclins A, B, D, and E, and CDKs 1 and 2, compared to normal tissues. This upregulation underscores the high cell cycle activity that drives tumor proliferation in HNSC, making cell cycle regulators attractive therapeutic targets. Gene set enrichment analysis further supports this by revealing that these upregulated genes are significantly associated with pathways controlling the G1/S and G2/M transitions, which are critical checkpoints that facilitate the rapid proliferation observed in OSCC. Our findings align with previous research indicating that cell cycle dysregulation is a hallmark of HNSC and other aggressive cancers, where overactive Cyclins and CDKs support unchecked cell division and tumor progression. By demonstrating that Dinaciclib downregulates these overexpressed cyclins and CDKs in OSCC cell lines, we highlight its potential to disrupt key cell cycle transitions, specifically at the G1/S and G2/M checkpoints, thus limiting OSCC cell proliferation. Importantly, Dinaciclib’s inhibition of CDKs and cyclins, as evidenced in this study, directly targets the G2/M and G1/S transitions (Figure 6). This dual inhibition strategy not only arrests cell cycle progression but also primes tumor cells for apoptosis, as evidenced by increased levels of cleaved-caspase-3 and cleaved-PARP. These results support the growing interest in CDK inhibitors as therapeutic agents in cancers with high cell cycle dependency. While other studies have explored the effectiveness of CDK inhibitors in breast and lung cancers [18,37,38,39], our findings contribute new insights into the utility of Dinaciclib in OSCC, a relatively unexplored area. The current results encourage further exploration of Dinaciclib as a monotherapy or in combination with other treatments to maximize its therapeutic potential in OSCC.

Our initial experiments showed that Dinaciclib significantly inhibits OSCC cell proliferation in a dose-dependent manner, yielding pronounced cytotoxic effects across all cell lines tested. These findings align with prior studies in other cancer types, including breast, pancreatic, and hematologic cancers, in which Dinaciclib’s inhibition of CDKs 1, 2, 5, and 9 effectively suppressed tumor cell proliferation and induced cytotoxicity [18,19,20]. This effect on OSCC cells highlights the relevance of targeting CDKs in this carcinoma, as dysregulated CDK signaling is known to contribute to unchecked cell division and tumor progression in OSCC. By inhibiting CDKs 1 and 2, which are crucial for the G1/S and G2/M transitions, Dinaciclib likely impedes the ability of OSCC cells to complete DNA replication and mitosis, thus reducing their proliferative potential.

Flow cytometry and Western blot analyses revealed that Dinaciclib induces distinct cell cycle arrest profiles across the three OSCC cell lines, with Ca9-22 cells predominantly arrested in the S phase and OECM-1 and HSC-3 cells in the G2/M phase. This variation may be due to intrinsic differences in the molecular profiles of these cell lines, suggesting that Dinaciclib’s impact on cell cycle regulation may be influenced by cellular context. Nonetheless, the consistent reduction in key regulators of the cell cycle, including Cyclins A, B, D, and E, supports Dinaciclib’s broad ability to inhibit progression through both the G1/S and G2/M checkpoints. These findings highlight Dinaciclib’s potential as a therapeutic agent capable of halting OSCC cell cycle progression by directly targeting multiple CDK–cyclin complexes, which are frequently dysregulated in OSCC.

Typically, CDK inhibition is associated with an increase in CDK inhibitors as part of the cell’s response to halt progression through the cell cycle. However, the observed downregulation of p21 and p27 in all OSCC cell lines treated with Dinaciclib suggests a unique and complex regulatory mechanism. It is possible that Dinaciclib’s inhibition of CDKs 1, 2, 5, and 9 leads to downstream effects on transcription factors or signaling pathways responsible for the maintenance of p21 and p27, such as p53, which is frequently altered in OSCC [40]. Rather than contributing to cell cycle arrest, the reductions in p21 and p27 may indicate a broader disruption of cellular homeostasis, thereby sensitizing cells to apoptosis [41]. This hypothesis is supported by our finding that Dinaciclib treatment led to increased sub-G1 populations and the upregulation of cleaved-caspase-3 and cleaved-PARP in all three OSCC cell lines. The reduced levels of p21 and p27 might hinder the cells’ ability to initiate DNA repair mechanisms, effectively rendering them more susceptible to apoptosis in response to cellular stress induced by Dinaciclib [42,43]. Thus, while p21 and p27 are traditionally viewed as protective regulators that halt cell cycle progression, their downregulation in this context may accelerate apoptosis, highlighting an alternative pro-apoptotic mechanism associated with Dinaciclib in OSCC.

Dinaciclib’s induction of apoptosis, as evidenced by increased levels of cleaved-caspase-3 and cleaved-PARP, underscores its therapeutic promise beyond cell cycle arrest alone. Caspase-3 activation is a hallmark of apoptosis, and its upregulation, alongside cleaved-PARP, suggests that Dinaciclib not only halts OSCC proliferation but actively promotes cell death through apoptotic pathways. This dual mechanism of action, combining cell cycle arrest and apoptosis induction, is particularly valuable in the context of OSCC, which is known for its resistance to conventional therapies [44,45,46,47]. Our findings demonstrate that Dinaciclib effectively induces cell cycle arrest at both the G1/S and G2/M transitions in OSCC cell lines, primarily by inhibiting key cyclin-dependent kinases (CDKs). However, it has been suggested that cancer cells possess the ability to adapt to CDK inhibition by activating compensatory signaling pathways, which can undermine the long-term efficacy of CDK-targeted therapies. Several studies have shown that upon CDK inhibition, cancer cells may upregulate alternative survival pathways to maintain proliferation and evade apoptosis. Notably, the PI3K/AKT/mTOR and MAPK/ERK signaling cascades are frequently activated as compensatory mechanisms [48,49]. The PI3K/AKT pathway promotes cell survival, growth, and metabolic adaptation, potentially counteracting the antiproliferative effects of Dinaciclib [50]. Similarly, the MAPK/ERK pathway can drive cell cycle progression independent of CDK activity, contributing to therapeutic resistance [51].

In addition to these pathways, the upregulation of Cyclin E–CDK2 complexes and the activation of CDK-independent transcription factors such as MYC can support cell cycle re-entry [52]. Cancer cells may also enhance the expression of anti-apoptotic proteins, including MCL-1 and BCL-2, which are known to confer resistance to CDK inhibitors by suppressing apoptotic signaling [53]. These compensatory mechanisms highlight the plasticity of cancer cells and underscore the need for combination therapies that target both CDKs and alternative survival pathways.

Our results align with those of recent studies demonstrating Dinaciclib’s apoptotic effects in various cancers. For instance, research on Dinaciclib in pancreatic cancer revealed that its apoptotic effects were mediated through both intrinsic and extrinsic pathways, and similar findings were reported in breast cancer models [18,37,38,39]. The ability of Dinaciclib to induce apoptosis in OSCC cells likely enhances its therapeutic efficacy, making it a promising candidate for clinical evaluation, particularly in OSCC patients with treatment-resistant tumors. In addition to its efficacy in OSCC cells, Dinaciclib has been shown to exhibit selective toxicity toward cancer cells while sparing normal cells, largely due to differential CDK dependencies. Cancer cells rely heavily on CDKs like CDK1, CDK2, and CDK9 for uncontrolled proliferation, whereas normal cells maintain cell cycle control with minimal CDK activity, making them less susceptible to Dinaciclib-induced cytotoxicity. While our study primarily focuses on the short-term efficacy of Dinaciclib in OSCC cell lines, it is crucial to consider its toxicological profile and the potential for resistance to develop, especially in the context of long-term clinical use. As a potent CDK inhibitor, Dinaciclib has shown promising anti-cancer activity; however, clinical studies have reported adverse effects such as neutropenia, gastrointestinal disturbances, fatigue, and hepatotoxicity, which may limit its therapeutic effect [54,55]. These toxicities are largely attributed to its broad inhibition of CDKs involved in normal cell cycle regulation, highlighting the need for careful dose optimization in clinical applications. Additionally, prolonged exposure to Dinaciclib may lead to the development of adaptive resistance mechanisms [56,57]. Emerging evidence suggests that cancer cells can activate compensatory signaling pathways, such as the PI3K/AKT and MAPK pathways, to bypass CDK inhibition and sustain proliferation. Moreover, alterations in apoptotic regulators, the upregulation of anti-apoptotic proteins (e.g., MCL-1), and mutations in CDK-related genes have been implicated in resistance to CDK inhibitors [21,58,59,60]. In addition, it is well documented that Dinaciclib exhibits its highest efficacy in actively cycling cells due to its increased reliance on CDK activity for proliferation [61]. On the other hand, serum starvation induces a quiescent state (G0 phase), significantly reducing CDK activity and potentially diminishing the effectiveness of CDK inhibitors like Dinaciclib [25,62]. This suggests that Dinaciclib’s antiproliferative effects may be less pronounced in non-dividing or slowly proliferating cells.

Overall, Dinaciclib offers distinct therapeutic potential compared to conventional OSCC treatments such as surgery, radiotherapy, and cisplatin-based chemotherapy. While cisplatin induces DNA damage to trigger apoptosis, its effectiveness is often limited by significant toxicity and the development of drug resistance [59]. In contrast, Dinaciclib targets key cell cycle regulators (CDK1, CDK2, CDK5, and CDK9), inducing cell cycle arrest and apoptosis through transcriptional suppression, making it effective even in cisplatin-resistant cells [59]. Moreover, Dinaciclib shows promise as a combination therapy, potentially enhancing the efficacy of chemotherapy and radiotherapy by impairing DNA repair mechanisms, increasing radiosensitivity, and overcoming resistance pathways. This combination approach may also allow for dose reductions in conventional therapies, minimizing systemic toxicity while improving treatment outcomes. Furthermore, although our study is based on in vitro models, we recognize the critical role of the tumor microenvironment (TME) and immune modulation in influencing therapeutic outcomes. Emerging evidence suggests that Dinaciclib can modulate the TME by affecting immune checkpoint molecules, potentially enhancing antitumor immunity [63,64]. Additionally, Dinaciclib may reduce immunosuppressive signaling pathways and impact the behavior of tumor-associated fibroblasts (TAFs), leading to alterations in the extracellular matrix (ECM) that could improve drug penetration and efficacy [65]. By influencing both cancer cell-intrinsic mechanisms and the TME, Dinaciclib holds promise not only as a direct antiproliferative agent but also as a modulator of the broader tumor ecosystem.

Our findings contribute to the current literature by providing novel insights into Dinaciclib’s mechanism of action in OSCC, a malignancy for which limited therapeutic options exist. The observed effects of Dinaciclib on both cell cycle arrest and apoptosis highlight its unique potential to address two critical hallmarks of cancer: uncontrolled proliferation and resistance to cell death. Moreover, the distinct response profiles in Ca9-22, OECM-1, and HSC-3 cells suggest that Dinaciclib may be effective across a range of OSCC subtypes, underscoring its broad applicability.

## 4. Materials and Methods

### 4.1. Cell Culture

Three human OSCC cell lines, Ca9-22, OECM-1, and HSC-3, were kindly gifted by Dr. Shun-Fa Yang at the Department of Medical Research, Chung Shan Medical University Hospital, Taichung, Taiwan. Cells were maintained in RPMI-1640 culture medium (Gibco, Carlsbad, CA, USA), supplemented with 10% fetal bovine serum (FBS; Gibco, Grand Island, NY, USA), and penicillin-streptomycin (P/S) at concentrations of 100 IU/mL and 100 μg/mL, respectively (Sigma-Aldrich, St. Louis, MO, USA), along with 2 mM L-glutamine, 1.5 g/L sodium bicarbonate (NaHCO_3_) (Sigma-Aldrich, St. Louis, MO, USA), 10 mM HEPES (Sigma-Aldrich, St. Louis, MO, USA), and 1 mM sodium pyruvate (Sigma-Aldrich, St. Louis, MO, USA). Cells were passaged every 3 days to maintain exponential growth and used for experiments at 70–80% confluency.

### 4.2. Drug Treatment

Dinaciclib was prepared as previously described [66]. In brief, Dinaciclib was prepared as a 100 µM stock solution in DMSO and diluted in culture media to final concentrations of 6.25 nM, 12.5 nM, and 25 nM. Control cells were treated with an equivalent concentration of DMSO (≤0.1%). In addition, the IC_50_ values for each OSCC cell line were tested and are presented in Table 1. The selected concentrations of Dinaciclib fall within the nanomolar range commonly reported to exert potent antiproliferative and pro-apoptotic effects in various cancer models. Pharmacokinetic studies indicate that plasma concentrations of Dinaciclib in patients typically range from 10 to 30 nM following standard dosing, aligning closely with the concentrations used in our experiments. The use of 6.25 nM allowed us to assess the drug’s efficacy at sub-therapeutic levels, while 25 nM reflects the upper limit of clinically achievable exposure [54]. This concentration range enabled us to capture dose-dependent effects and provided translational relevance to potential therapeutic applications in OSCC.

### 4.3. Cell Viability Assay

Cell viability assay was performed as previously described [67]. In brief, cell viability was measured using an MTT assay. OSCC cells were seeded in 96-well plates (5 × 10^4^ cells/well) and treated with increasing concentrations of Dinaciclib for 24 h. After the treatment of cells in a dose-dependent manner in the 96-well plates, the culture medium was removed, and the MTT solution was diluted in the appropriate medium without serum (0.5 mg/mL) in each well. The plate was then incubated at 37 °C for 3 h, allowing viable cells to convert MTT into formazan crystals. DMSO was used to solubilize the formazan crystals. Absorbance was measured at 570 nm using a microplate reader.

### 4.4. Cell Cycle Analysis

Cell cycle distribution was analyzed using flow cytometry. The OSCC cells were treated with Dinaciclib for 24 h, harvested, and fixed in 70% ethanol overnight. The cells were then stained with a propidium iodide (PI) solution containing RNase A and analyzed on a Sony flow cytometer (SA3800). The percentages of cells in the G1, S, and G2/M phases were calculated using software SA3800 V.2.1.0.

### 4.5. Western Blotting

Protein expression levels were assessed via Western blotting. Treated and untreated OSCC cells were lysed in RIPA buffer containing protease and phosphatase inhibitors. Protein concentrations were determined using a BCA protein assay. Equal amounts of protein (30 μg) were separated via SDS-PAGE and transferred to PVDF membranes. The membranes were blocked with 5% non-fat milk and incubated with primary antibodies against Cyclins A, B, D, and E, CDK1, CDK2, cleaved-caspase-3, cleaved-PARP, p21, and p27 (all obtained from Cell Signaling Technology, Danvers, MA, USA) overnight at 4 °C. The membranes were incubated with HRP-conjugated secondary antibodies, and bands were visualized using an ECL detection reagent.

### 4.6. Statistical Analysis

Statistical analysis and graph generation were conducted using GraphPad Prism software (v. 10.2.1). Data are presented as means ± SD (standard deviation). The unpaired Student’s *t*-test was utilized to compare the two groups. A *p*-value < 0.05 was considered statistically significant.

## 5. Conclusions

In conclusion, our study demonstrates that Dinaciclib effectively disrupts cell cycle progression and induces apoptosis in OSCC cells through a multifaceted mechanism involving the downregulation of critical cyclins, CDKs, and the CDK inhibitors p21 and p27. This dual mode of action not only halts proliferation but also sensitizes cells to apoptosis, providing a potent therapeutic strategy for OSCC. Further in vivo studies and clinical trials are warranted to explore Dinaciclib’s potential in OSCC treatment, particularly in cases of resistance to standard therapies.

## Figures and Tables

**Table 1:** Effects of Dinaciclib on cell cycle phase distribution in Ca9-22 OSCC cells.

Dina (nM)	0	6.25	12.5	25
G1	76.26 ± 0.81	66.84 ± 1.76 **	38.46 ± 0.8 ***	24.37 ± 0.91 ***
S	3.03 ± 0.07	4.34 ± 0.27 **	22.67 ± 1.37 ***	11.74 ± 0.63 ***
G2/M	19.37 ± 0.67	26.22 ± 1.54 *	19.2 ± 0.98	1.78 ± 0.21 ***
sub-G1	0.8 ± 0.05	1.24 ± 0.02 **	10.97 ± 1.15 ***	52.7 ± 0.46 ***
* compared to 0.				

The cell cycle phase distribution (G1, S, G2/M, and sub-G1) of Ca9-22 cells treated with increasing concentrations of Dinaciclib (0, 6.25, 12.5, and 25 nM) for 24 h. Values are expressed as mean ± SD, with statistical significance denoted relative to the untreated control (0 nM). Asterisks indicate significant changes in cell cycle distribution, with *p* < 0.05 (*), *p* < 0.01 (**), and *p* < 0.001 (***). Dinaciclib induces S phase and sub-G1 accumulation at higher concentrations, which is consistent with cell cycle arrest and apoptosis.

**Table 2:** Effects of Dinaciclib on cell cycle phase distribution in OECM-1 OSCC cells.

Dina (nM)	0	6.25	12.5	25
G1	73.47 ± 0.84	68.49 ± 0.79 *	61.78 ± 1.03 ***	72.61 ± 0.68
S	6.37 ± 0.61	7.88 ± 0.29	11.42 ± 0.61 **	5.31 ± 0.07
G2/M	19.14 ± 0.15	22.67 ± 0.53 **	25.66 ± 0.45 ***	14.86 ± 0.13 ***
sub-G1	0.7 ± 0.07	0.59 ± 0.08	0.57 ± 0.06	6 ± 0.56 ***
* compared to 0.				

The impact of Dinaciclib on cell cycle distribution in OECM-1 cells across different phases (G1, S, G2/M, and sub-G1) at concentrations of 0, 6.25, 12.5, and 25 nM. Data are represented as mean ± SD values, with statistical significance compared to the control indicated by asterisks (* *p* < 0.05, ** *p* < 0.01, *** *p* < 0.001). Dinaciclib treatment leads to a dose-dependent increase in G2/M phase cells at intermediate concentrations, along with a significant increase in sub-G1 phase cells at the highest dose, indicating apoptotic induction.

**Table 3:** Effects of Dinaciclib on cell cycle phase distribution in HSC-3 OSCC cells.

Dina (nM)	0	6.25	12.5	25
G1	66.43 ± 0.96	49.3 ± 0.73 ***	54.26 ± 0.33 ***	48.81 ± 0.3 ***
S	7.41 ± 0.16	11.9 ± 0.18 ***	6.44 ± 0.2 *	12.67 ± 0.84 **
G2/M	21.41 ± 0.42	31.39 ± 0.83 ***	32.89 ± 0.03 ***	28.49 ± 0.78 **
sub-G1	0.44 ± 0.02	0.56 ± 0.06	0.66 ± 0.04 *	1.69 ± 0.15 **
* compared to 0.				

The distribution of HSC-3 cells across cell cycle phases (G1, S, G2/M, and sub-G1) following treatment with Dinaciclib at concentrations of 0, 6.25, 12.5, and 25 nM. Results are given as mean ± SD, with statistical comparisons to an untreated control denoted by asterisks (* *p* < 0.05, ** *p* < 0.01, *** *p* < 0.001). Dinaciclib causes significant G2/M arrest and a modest increase in sub-G1 phase cells at the highest concentration, highlighting its effect on cell cycle arrest and apoptosis in HSC-3 cells.

## Data Availability

Data are contained within the article.
